# Bioactive Molecules from the Exoskeleton of *Procambarus clarkii*: Reducing Capacity, Radical Scavenger, and Antitumor and Anti-Inflammatory Activities

**DOI:** 10.3390/biom14121635

**Published:** 2024-12-20

**Authors:** Francesco Longo, Francesca Di Gaudio, Alessandro Attanzio, Laura Marretta, Claudio Luparello, Serena Indelicato, David Bongiorno, Giampaolo Barone, Luisa Tesoriere, Ilenia Concetta Giardina, Giulia Abruscato, Manuela Perlotti, Lucie Branwen Hornsby, Vincenzo Arizza, Mirella Vazzana, Federico Marrone, Aiti Vizzini, Chiara Martino, Dario Savoca, Vinicius Queiroz, Antonio Fabbrizio, Manuela Mauro

**Affiliations:** 1 Department of Biological, Chemical and Pharmaceutical Sciences and Technologies (STEBICEF), University of Palermo, 90123 Palermo, Italy; francesco.longo03@unipa.it (F.L.); alessandro.attanzio@unipa.it (A.A.); laura.marretta02@unipa.it (L.M.); claudio.luparello@unipa.it (C.L.); serena.indelicato@unipa.it (S.I.); david.bongiorno@unipa.it (D.B.); giampaolo.barone@unipa.it (G.B.); luisa.tesoriere@unipa.it (L.T.); ileniaconcetta.giardina@unipa.it (I.C.G.); manuela.perlotti@community.unipa.it (M.P.); lbhornsby@gmail.com (L.B.H.); vincenzo.arizza@unipa.it (V.A.); mirella.vazzana@unipa.it (M.V.); federico.marrone@unipa.it (F.M.); aiti.vizzini@unipa.it (A.V.); chiara.martino@unipa.it (C.M.); dario.savoca@unipa.it (D.S.); 2 Department PROMISE, University of Palermo, Piazza delle Cliniche, 2, 90127 Palermo, Italy; francesca.digaudio@unipa.it; 3 National Biodiversity Future Center (NBFC), Piazza Marina 61, 90133 Palermo, Italy; 4 Departamento de Fisiologia, Instituto de Biociências, Universidade de São Paulo, São Paulo 05508-090, Brazil; vinicius_ufba@yahoo.com.br; 5 Department of Theoretical and Applied Sciences (DiSTA), University e Campus, 22060 Novedrate, Italy; antonio.fabbrizio@uniecampus.it

**Keywords:** astaxanthin, chitosan, crustacean, freshwater, invertebrates, phenolic compounds, antioxidant

## Abstract

This study evaluates, for the first time, the reducing capacity, radical scavenger activity, and *in vitro* antitumor and anti-inflammatory effects of chitosan, astaxanthin, and bio-phenols extracted from the exoskeleton of Sicilian *Procambarus clarkii*, the most widespread species of invasive crayfish in the Mediterranean region. Among the extracted compounds, astaxanthin exhibited the highest antioxidant activity in all assays. Chitosan and polyphenols demonstrated reducing and radical scavenging activity; chitosan showed significant ferric ion reducing capacity in the FRAP test, while bio-phenolic compounds displayed notable radical scavenging activity in the DPPH and ABTS assays. Both astaxanthin and polyphenols showed dose-dependent cytotoxicity on two different cancer cell lines, with IC50 values of 1.45 µg/mL (phenolic extract) and 4.28 µg/mL (astaxanthin extract) for HepG2 cells and 2.45 µg/mL (phenolic extract) and 4.57 µg/mL (astaxanthin extract) for CaCo-2 cells. The bio-phenolic extract also showed potential anti-inflammatory effects *in vitro* by inhibiting nitric oxide production in inflamed RAW 264.7 macrophages, reducing the treated/control NO ratio to 77% and 74% at concentrations of 1.25 and 1.5 μg/mL, respectively. These results suggest that *P. clarkii* exoskeletons could be a valuable source of bioactive molecules for biomedical, pharmaceutical, and nutraceutical application while contributing to the sustainable management of this invasive species.

## 1. Introduction

Crustaceans are a valuable source of bioactive molecules [1]. One extractable product from their exoskeletons is chitin [2,3,4], the second most abundant biopolymer in nature after cellulose [5]. Chitin is a polysaccharide composed of N-acetylglucosamine units linked by a β-1,4 glycosidic bond; it can be deacetylated to obtain chitosan, another polysaccharide with multiple applications [4]. This biopolymer is used in wastewater treatment, exploiting its ability to chelate metal cations [6], in the agricultural sector, using its antimicrobial properties to reduce applications of pesticides [7], and in biomedical bandages to accelerate wound healing by promoting erythrocyte adhesion and fibrinogen adsorption [8,9]. Chitosan also serves as an excipient due to its film-forming, viscosifying, and mucoadhesive properties [10], as a carrier in controlled-release pharmaceutical formulations for targeted drug delivery [11], and as a bioactive molecule thanks to its antitumor, antimicrobial, anti-inflammatory, and antioxidant properties [12,13]. Astaxanthin is another interesting biomolecule that can be extracted from the exoskeletons of crustaceans; it is one of the most powerful antioxidants in nature [14,15,16]. This molecule is not biosynthesized by crustaceans themselves but by algae they can feed on, such as *Haematococcus pluvialis* [17].

Astaxanthin is a carotenoid characterized by a series of conjugated double bonds, which are responsible for both its high antioxidant activity and its characteristic intense red color. It is this carotenoid that contributes to the typical pinkish-red color of crustaceans and fish, such as salmon and trout [17]. Its powerful antioxidant activity is used in the cosmetics industry to produce anti-aging creams and lotions, as well as in supplements and nutraceutical products. Furthermore, the Food and Drug Administration and the European Commission have approved the use of astaxanthin as a food additive in animal and fish feed [18,19,20]. Crustacean exoskeletons also contain phenolic compounds, another class of molecules with notable antioxidant activity [21]. These molecules can be divided into phenolic acids, stilbenes, and flavonoids (flavanols, flavones, flavanones, isoflavones, flavan-3-ols, and anthocyanins) based on their structure [22]. In addition to being powerful antioxidants, these compounds exhibit a large range of biological activities, such as antitumor, anti-inflammatory, antihypertensive, antidiabetic, antimicrobial, antiviral, algicidal, antifungal, and insecticidal activities [23,24]. Although plant-derived phenolic compounds have been the most studied in the past, the phenolic composition of aquatic organisms, including, in particular, crustaceans, has recently enjoyed more investigation [21,25].

The red swamp crayfish *Procambarus clarkii* is the most widespread crayfish species in the world. It is an alien and invasive species native to the inland waters of south–central United States and northeastern Mexico [26,27,28]. Due to its biological characteristics, *P. clarkii* poses a significant threat to freshwater biodiversity [29]. It is an extremely opportunistic species capable of feeding on a wide range of organic material, both animal-and plant-based, including plants, algae, insects, eggs, and the larvae and juvenile stages of fish and amphibians, thus endangering native species [27]. The widespread presence of this species is attributed to both its biological characteristics and human activity, as *P. clarkii* is highly valued in both aquaculture and aquariums, the two main causes of voluntary or accidental introduction of this species into nature [30,31,32]. Once introduced into the wild, the species spreads rapidly due to its characteristic resistance to a wide range of water temperatures and salinity levels, together with its high reproductive rate (e.g., two breeding events per year, with females spawning up to 600 eggs) [33]. Furthermore, *P. clarkii* can breathe out of water for over 10 h and disperse over land, covering distances of more than 1 km. This enables them to move between water bodies, allowing them to colonize even isolated water bodies, such as pools or ponds [34,35]. Given these characteristics, *P. clarkii* has been listed as an invasive alien species of community importance since 2016 [36]. Under EU Regulation 1143/14, a series of prohibitions and intervention obligations are outlined, primarily aimed at preventing new releases into the wild, controlling and containing invasions in already colonized areas, and limiting its impact on local ecosystems. In recent decades, *P. clarkii* has made its way to the Mediterranean Basin, where it is considered an invasive species [37].

Despite the significant damage *Procambarus clarkii* has inflicted on both biodiversity and the economy in colonized regions, numerous studies have demonstrated that this invasive species can be utilized as a source of bioactive molecules, with important applications in the biotechnological, biomedical, and pharmaceutical sectors [2,3,4,15,16,38,39,40]. In 2017, Elkhodary et al. [37] determined the total phenolic and flavonoid contents in specimens collected from the River Nile; however, the phenolic profile of the exoskeleton of this species remains uncharacterized.

The aim of this study was to determine the composition of the main bioactive molecules of the exoskeleton of Sicilian *Procambarus clarkii* (chitosan, astaxanthin, phenolic compounds) and to evaluate their antioxidant activity. A qualitative and quantitative analysis was conducted to characterize the phenolic compounds. Astaxanthin and polyphenols were subsequently tested for their cytotoxic effects on two human cancer cell lines—HepG2 (hepatocarcinoma) and CaCo-2 (colorectal carcinoma)—and for their ability to counteract nitric oxide production in inflamed RAW 264.7 murine macrophages. This study represents the first comparison of the antioxidant, antitumoral, and anti-inflammatory activities of these bioactive molecules. The findings of this study help identify new sources of bioactive molecules by using an alien and invasive species harmful to the biodiversity and economy of freshwater ecosystems and for which effective eradication methods are currently being explored.

## 2. Materials and Methods

### 2.1. Samples

In June 2023, 120 specimens of *Procambarus clarkii* (total length 10.14 ± 0.98 cm and weight 30.23 ± 9.30 g) were collected from Lake Rosamarina (coordinates: 37°54′20.4″ N 13°36′33.9″ E), an artificial reservoir created by damming the “San Leonardo” river in the municipality of Caccamo in the province of Palermo (Italy) [41]. The animals were captured using cylindrical traps and immediately transported to the STEBICEF Department at the University of Palermo. After being weighed and measured, the animals were euthanized by placing them on ice for at least an hour. The exoskeleton was then sampled from each specimen.

### 2.2. Exoskeleton Flour Production

The exoskeletons were divided into three pools, each consisting of the exoskeletons from 40 specimens, and each pool was dried in an oven at 60 °C for 48 h and then ground and sieved to obtain fine powders with uniform particle sizes. The resulting powders were stored at room temperature. The percentage of humidity of the exoskeleton for each pool was calculated using the following formula:

(1) $$Umidity\;\%=100-\left(\frac{dry\;weight\times 100}{wet\;weight}\right)$$

where *dry weight* represents the weight of the exoskeletons after reaching a constant weight over time following oven drying, while *wet weight* refers to the weight of the exoskeletons prior to drying.

### 2.3. Chitosan Extraction and FT-IR Characterization

Chitosan was extracted from each exoskeleton flour pool using a modified version of the protocol previously published by Mauro et al. [13]. This protocol includes 4 key steps: deproteinization, demineralization, decolorization, and deacetylation. During deproteinization, the exoskeleton powders were suspended in a 3% NaOH solution (1:10 *w*/*v*) for two hours at 65–70 °C to release the protein [42]. The sample was then washed three times using distilled water and dried overnight at 60 °C. This washing procedure was repeated at the end of each step. During demineralization, the sample was treated with a 1 M HCl solution at a 1:15 *w*/*v* for 1 h at room temperature. The demineralized sample then underwent decolorization through suspension in a 3% NaClO solution (1:10 *w*/*v*) for 2 h at room temperature. Following further washing and drying, chitin was obtained. To obtain chitosan, the chitin was deacetylated using a 50% NaOH solution (1:15 *w*/*v*) for 48 h at 90 °C. Subsequently, the chitosan was purified in a 0.5 M acetic acid solution (1:100 *w*/*v*). The resulting dispersion was filtered. A 10% NaOH solution (10%) was added until pH 8 was reached. The dispersion was then centrifuged at 4000 rpm for 10 min at room temperature. The pellet was collected and washed three times with ethanol 70%. Finally, the pure chitosan was dried at 60 °C for 18 h, weighed, and stored for subsequent testing.

The final yield of chitosan was calculated using the following formula:

(2) $$Yield(\%)=\frac{Dry\;chitosan\;weight\times 100}{Exoskeleton\;flour\;weight}$$

The chitosan was characterized using Fourier Transform Infrared (FTIR) spectroscopy. The sample was prepared with KBr, and the spectrum was recorded in the range of 4000–300 cm^−1^ using a Jasco FT/IR 420 spectrometer. Transmittance (T) values at 1320 cm^−1^ and 1420 cm^−1^, converted to absorbance (A) values, were used to calculate the percentage degree of acetylation (DA) of chitosan using the following equation [43]:

(3) $$DA\%=\frac{\left[\left(A1320/A1420\right)-0.3822\right]}{0.03133}$$

Subsequently, the percentage of the degree of deacetylation (DDA) of chitosan was calculated using the following formula:

(4) DDA%=100−DA%

### 2.4. Astaxanthin Ultrasound-Assisted Extraction

Astaxanthin was extracted using an ultrasound-assisted extraction technique based on a modified protocol by Hu et al. [44]. For each exoskeleton flour pool, a known amount of powder was suspended in absolute ethanol in a *w*/*v* ratio of 1:4. The suspension was gently stirred in the dark at room temperature for 1 h and then placed on ice and subjected to ultrasonic treatment. Ultrasonication was performed using a VC505 ultrasonic generator (Sonics & Materials Inc., Newtown, CT, USA) operating at a frequency of 20 kHz and a power output of 300 W for 30 min. The sample was subsequently centrifuged at 1500× *g* and 4 °C for 10 min, after which the supernatant was collected and filtered. A second extraction was conducted on the solid residue. Extracts were stored in the dark at 2–8 °C until analysis.

#### Astaxanthin Determination Through HPLC-HRMS

The HPLC-HRMS analyses of ethanolic extracts were conducted using a UHPLC Ultimate 3000 RS (Dionex, Waltham, MA, USA) coupled with EXPLORIS 120 (ThermoFisher, San Jose, CA, USA). For chromatographic separation, a Hypersil Gold column (50 × 2.1 mm, 1.9 µm) was maintained at a temperature of 30 °C. The LC method used the following mobile phases: (A) purified water (LC–MS-grade, Sigma-Aldrich, Darmstadt, Germany) with 0.1% formic acid (LC–MS-grade, Sigma-Aldrich, Darmstadt, Germany) and (B) acetonitrile (LC–MS-grade, Sigma-Aldrich, Darmstadt, Germany). The flow rate was set at 300 µL/min. The gradient program was as follows: 0–8.0 min, 60% B, 8.0–13.0 min, linear increase to 100% B, 13.0–17.0 min, hold at 100% B, 17.0–17.01 min, linear decrease to 60% B, and 17.1–20 min, hold at 60% B. The injection volume was 10 μL.

The mass spectrometer was equipped with a heated electrospray (HESI) ionization source. The LC/MS–MS experiments were conducted in positive ion mode. The mass spectrometry analyses were performed under the following experimental conditions: HESI (+), spray voltage (static) of 3500 V, ion transfer tube temperature of 350.00 °C, auxiliary gas pressure of 15 a.u., sheath gas pressure of 50 psi, auxiliary 10 a.u., and S-Lens RF Level 70.00 V. Full MS experiments included Microscans 1, a resolution of 17,500, an AGC target of 5 × 106, a maximum IT of 200 ms, a number of scan ranges of 1, a scan range of 500 to 700 *m*/*z*, and a profile spectrum data type. The orbitrap resolution was 60,000. The product ion scan conditions were RF lens (%) of 70, AGC target set to standard, and positive polarity.

In MS/MS spectra, the precursor ion was set to 597.3938 (*m*/*z*), the formula was C_40_H_52_O_4_, the isolation window was 4.0 *m*/*z*, the collision energy (%) was set at 10, 25, and 35 volts, the collision gas was N2, the resolution was 15,000, and the AGC target was standard.

To obtain an identification and quantification of astaxanthin in ethanolic extracts, an external calibration method was used. Standard solutions of astaxanthin (>70%, from Blakeslea Trispora, Sigma Aldrich, Saint Louis, MO, USA) in ethanol were prepared at concentrations of 1000 ppb, 500 ppb, 100 ppb, and 10 ppb. The equation of the calibration curve was Y = 2.85E + 04X − 5.95E + 05, with the linearity correlation coefficient (R^2^) equal to 0.99.

Prior to analysis using the HPLC-HRMS instrument, each sample was prepared using CLARIFY-PTFE 0.45 µm (13 mm) filters and diluted to 1:100 with methanol.

### 2.5. Extraction of Phenolic Compounds and Total Phenol Content (TPC)

Phenolic compounds were extracted by suspending 1 g of each pool of exoskeleton flour in 4 mL of 80% methanol (*v*/*v*) in the dark at room temperature, with agitation for 1 h. The suspension was then sonicated at 40 °C in a water bath for 30 min using ultrasound equipment and centrifuged at 3700× *g* for 10 min. A second extraction was performed on the solid residue. The supernatants were filtered through a 0.45 μm filter (Millipore, Billerica, MA, USA) using a Büchner funnel and stored in the dark at 2–8 °C until subsequent analysis.

The total phenol content (TPC) was determined using the Folin–Ciocalteu reaction, which is based on the reduction of phosphotungstic–phosphomolybdic acid (Folin–Ciocalteu’s reagent, Sigma-Aldrich, Saint Louis, MO, USA) to form blue reaction products in an alkaline solution [45].

The extracts were treated to remove methanol and then freeze-dried to remove residual water. The dried samples were solubilized in 5 mM of phosphate-buffered saline (PBS) at pH 7.4 before being used in the experimental analysis that followed [46]. Aliquots of samples (10–100 μL) were added to a final volume of 100 μL of water and mixed with 100 μL of Folin–Ciocalteu reagent for 5 min. Then, 3 mL of 2% sodium carbonate was added. The reaction mixture was incubated at room temperature in the dark for 60 min, and absorbance at 700 nm was measured using a Beckman DU640 spectrophotometer (Beckman, Milan, Italy) against a blank without samples. Quantification was performed using a gallic acid calibration curve (10–100 μg/mL), with the results expressed as gallic acid equivalents (GAE, milligrams/1 g of sample).

#### Determination of Phenolic Compounds Through HPLC-HESI-MS

Identification of phenolic compounds in methanolic extract of exoskeleton flours of *Procambarus clarkii* was partly based on the method described by Indelicato et al. [47] with modifications to develop an UHPLC/MS-MS protocol. LC–MS/MS analysis was carried out using an Ultimate 3000 instrument coupled to a TSQ Quantiva (Thermo Fisher Scientific, San José, CA, USA) triple-stage quadrupole mass spectrometer. Chromatographic separation was achieved using a C18 reversed-phase analytical column, Hypersil GOLD (2.1 × 50 mm, 1.9 μm particle size, Thermo Fisher Scientific), maintained at 30 °C, with an injection volume of 5 μL. Analyses employed a gradient created by combining mobile phase (A), purified water with 0.1% formic acid (LC–MS-grade, Sigma-Aldrich, Saint Louis, MO, USA), and mobile phase (B), methanol (LC–MS-grade, Sigma-Aldrich, Saint Louis, MO, USA), at a constant flow rate of 300 μL/min. The gradient program was as follows: 0–2 min, 5% B, 2–10 min, linear increase to 70% B, 10–12 min, linear increase to 100% B, 12–17 min, hold at 100% B, 17.0–17.1 min, linear decrease to 1% B, and 17.1–19 min, hold at 1% B. The mass spectrometer (QqQ) (Thermo Scientific, Brema, Germany) was equipped with a heated electrospray (HESI) ion source. LC/MS–MS experiments were acquired in negative ion mode and tuned using standard solutions of each analyte at 1 ppm in methanol. Mass spectrometry analyses were performed under the following experimental conditions: HESI (−), spray voltage (static) of 2500 V, auxiliary gas pressure of 10 a.u., sheath gas pressure of 50 psi, sweep gas of 1 a.u, ion transfer tube at 325 °C, vaporizer temperature at 350 °C, dwell time of 100 ms, Q1 resolution of 1 Da, Q3 resolution of 0.4 Da, and CID gas (Ar) at 2.0 mTorr. Selected Reaction Monitoring (SRM) was performed on the deprotonated molecules for each polyphenol ([M-H]^−^), with SRM transitions detailed in Table 1. The quantification method was based on the integration of all areas of the monitored transitions. Quantitation was performed using the following pure standards: Apigenin 7-Glucoside, Apigenin, Quercetin, gallic acid, L-Mandelic Acid, Chlorogenic Acid, hydroxycinnamic acid, Kaempferol, Caffeic Acid, Vanillic Acid, Catechin, rutin, coumaric acid, Syringic Acid, gentisic acid, ferulic acid, Luteonin, and Resveratrol.

**Table 1:** UHPLC-MS parameters and limit of quantification for phenolic compounds identified in methanolic extracts of *P. clarkii*.

	Precursor Ion (*m*/*z*) [M-H]^−^	Product Ion (*m*/*z*)	Collision Energy (V)	RF Lens (V)	LOD (µg/L)
Gallic Acid	169	79	24	101	30
	169	125	14	101	
Vanillic Acid	177	123	20	105	32
	177	152	20	105	
Ferulic Acid	193	134	15	99	22
	193	178	13	99	
Chlorogenic Acid	353	179	45	180	26
	353	191	45	180	
Catechin	289	203	20	147	28
	289	245	15	147	
Mandelic Acid	151	77	18	65	26
	151	107	10	65	
Gentisic Acid	153	108	22	90	24
	153	109	14	90	
Syringic Acid	197	153	12	100	23
	197	182	14	100	
Caffeic Acid	179	107	25	101	26
	179	135	16	103	
Trans-OH-Cynnamic	163	93	31	90	27
	163	119	14	90	
Rutin	609	271	60	299	26
	609	300	38	299	
Resveratrol	227	143	27	156	25
	227	185	20	156	
Apigenin-7Glu	433	269	20	123	24
	433	271	20	123	
Quercetin	301	151	18	166	25
	301	179	21	166	
Kaempferol	285	202	20	195	26
	285	239	29	195	
Hydroxytyrosol	153	95	21	97	25
	153	123	14	97	
Coumaric Acid	163	93	31	91	27
	163	119	13	91	
Luteolin	285	133	35	187	26
	285	175	26	187	
Apigenin	269	117	35	178	25
	269	151	25	178	

For the quantitation of phenolic compounds, an external calibration procedure was employed. A methanolic solution containing 5 ppm of each analytical standard was prepared. From this solution, five additional calibration solutions were prepared at concentrations of 1 ppm, 500 ppb, 250 ppb, 100 ppb, and 50 ppb for each analyte. The linearity correlation coefficient (R^2^) was 0.99. Data were evaluated using the Quan/Qual Browser Trace Finder (Thermo Fisher Scientific, San Jose, CA, USA). Each point on the calibration graph corresponded to the average of three independent injections. Limits of detection (LODs) and limits of quantification (LOQs) for each compound in the standard solutions were estimated through the blank signal and regression curve (average of five blanks injected between standards) collected in the same elution time window as the individual substance. The LOD concentration was calculated as the concentration, giving a signal corresponding to the sum of the blank signal and three times its standard deviation, while the LOQ concentration was calculated as the concentration, giving a signal corresponding to the sum of the blank signal and ten times its standard deviation.

### 2.6. Reducing Capacity and Radical Scavenging Test

#### 2.6.1. Sample Preparation

For the FRAP, DPPH, and ABTS assays, chitosan was dissolved in 2 M of acetic acid to create a 2% (*w*:*v*) solution; the ethanolic extract of astaxanthin was used as is, and the dehydrated phenolic extract was resuspended in 1 mL of 5 mM phosphate-buffered saline (PBS) at pH 7.4.

#### 2.6.2. Reducing Capacity Test

A Ferric Ion Reducing Antioxidant Power (FRAP) assay was conducted on chitosan, astaxanthin, and phenolic extracts, following the Saxena et al. [48] method, with minor modifications. Differing volumes of the samples were diluted with phosphate buffer 0.2 M (pH 6.6) to a final volume of 1 mL. Appropriately diluted samples were then mixed with 1 mL of 1% potassium ferricyanide and incubated at 50 °C for 20 min. Then, 1 mL of 10% trichloroacetic acid was added and mixed thoroughly using a vortex mixer. The resulting reaction mixture was centrifuged at 1000× *g* for 10 min. Aliquots of 0.5 mL of the supernatants were then mixed with 0.5 mL of distilled water and 0.1 mL of FeCl_3_ solution (0.1%). Finally, absorbance of the solution was measured at 700 nm. Ascorbic acid (0–100 μg/mL) was used as a reference compound, and the results were expressed as milligrams of ascorbic acid equivalents (AAE) per 1 g of dry sample.

#### 2.6.3. Radical Scavenging Activity Assay

A 2,2′-azinobis (3-ethylbenzothiazoline-6-sulfonic acid) (ABTS^•+^) radical cation was prepared following the method outlined by Re et al. [49] by reacting ABTS with potassium persulfate. Stock solutions consisted of 7.0 mM of ABTS solution and 140 mM of potassium persulfate solution. The working solution was created by combining 1 mL of the ABTS solution with 18 μL of the potassium persulfate solution and allowing them to react for 18 h in the dark at room temperature. The resulting solution was then diluted by mixing 900 μL of ABTS^•+^ solution with 100 μL of phosphate saline buffer to achieve an absorbance of approximately 0.700 units at 734 nm. Aliquots of adequately diluted sample (100 μL) were allowed to react with 900 μL of the ABTS^•+^ solution for 15 min in the dark, and the absorbance was subsequently measured at 734 nm.

2,2-Diphenyl-1-picrylhydrazyl (DPPH^•^) free radical scavenging activity was evaluated following the method described by Brand-Williams et al. [50]. Aliquots (100 μL) of appropriately diluted sample were added to 900 μL of DPPH^•^ ethanol solution (1 × 10^−4^ mol/L), and the absorbance was measured at 515 nm after 30 min of incubation at room temperature in the dark. The ABTS^•+^ and DPPH^•^ radical scavenging activities of the samples were compared with Trolox, the water-soluble analog of vitamin E, and the results were expressed as micromoles of Trolox equivalents (TE) per 1 g of samples.

### 2.7. In Vitro Antitumor and Anti-Inflammatory Activity

Antitumoral activity was evaluated on astaxanthin and phenolic compounds prepared as described in the previous section. HepG2 liver cancer cells and CaCo-2 colon cancer cells taken from laboratory stocks were grown in glutamine-containing DMEM medium (GibcoTM, Fisher Scientific, Segrate, Italy) supplemented with 10% heat-inactivated fetal bovine serum (Sigma-Aldrich, Saint Louis, MO, USA) and antibiotics (100 U/mL of penicillin and 100 g/mL of streptomycin; Capricorn Scientific GmbH, Ebsdorfergrund, Germany) at 37 °C under 5% CO_2_ in humidified air. The viability test was performed as already reported [51]. Cells in exponential growth were seeded at a concentration of 8000/well (HepG2) and 12,000/well (CaCo-2) in 96-well plates, allowed to adhere overnight, and cultured under control conditions or exposed to varying concentrations of either astaxanthin or polyphenol extract for 24 h. Astaxanthin was also tested 7 days after extraction to monitor its stability. After the addition of 3-(4,5-dimethylthiazol-2-yl)-2,5 diphenyl tetrazolium bromide (MTT; Merck, Milano, Italy) and cell solubilization, the absorbance of the dissolved formazan was measured using an automated microplate reader at l = 550 nm. The cell viability ratio in each experimental condition was determined as the ratio between the O.D. of the exposed cells and that of the controls. The half-maximal inhibitory concentrations (IC_50_) for astaxanthin and phenol extract were evaluated using ED50 PLUS V1.0 software, available online at https://www.sciencegateway.org/protocols/cellbio/drug/data/ed50v10.xls (accessed on 10 July 2024).

The ability of astaxanthin and polyphenol extracts to scavenge the NO radical was evaluated using the mouse macrophage-like cell line RAW 264.7, taken from laboratory stocks and cultured under the same conditions as the HepG2 and CaCo-2 cells. Before the assay, the effect of the extracts on cell viability was checked through an MTT assay, as described above, and the maximum non-inhibitory concentrations (MNICs) were quantitated to be used for the following assay. Cells in exponential growth were then seeded at the concentration of 150,000/well in 24-well plates, allowed to adhere overnight, and cultured in control conditions or exposed to the MNICs of either astaxanthin or polyphenol extract in the presence of LPS (0.1 μg/mL) for 24 h. The culture media from the control and the treated cells were collected and then transferred to a 96-well plate. Following the manufacturer’s recommendation, the absorbance of the samples was measured in an automated microplate reader at λ = 548 nm after 30 min of incubation with the Griess reagent (Biotium, Fremont, CA, USA) at room temperature. The NO release ratio in each experimental condition was determined as the ratio between the O.D. of the media from the exposed cells and that of the controls.

### 2.8. Statistical Analysis

All experiments were performed in at least triplicate (*n* ≥ 3). All data were presented as mean values ± standard deviation (SD) or standard error of the mean (s.e.m.; for cellular assays). Significant differences in the data were analyzed using one-way analysis of variance (ANOVA), followed by Tukey’s test, with a significance level of *p* < 0.05.

## 3. Results

### 3.1. Exoskeleton Yield and Humidity

Starting with 120 specimens of *Procambarus clarkii* weighing 3.627 kg, and after removing the abdominal muscle and cleaning the shells of soft tissue residues, a total weight of 3.186 kg of exoskeletons was obtained. This represents 87.84% of the total weight. The percentage of humidity of the exoskeleton was 66.61 ± 0.31%.

### 3.2. Chitosan Extraction and Characterization

**Figure 1:**
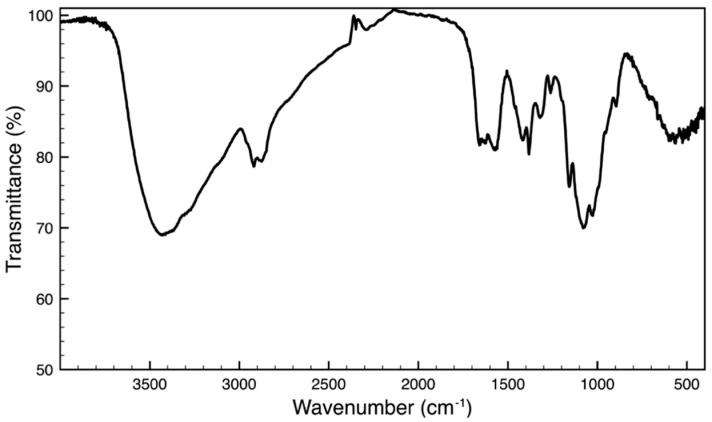
IR spectrum of chitosan extracted from *P. clarkii*.

The chitosan extraction yield was 12.3 ± 0.7%. The FTIR spectrum (Figure 1) revealed characteristic peaks corresponding to chitosan functional groups. In particular, the broad peak at 3433 cm^−1^ is attributed to the stretching of O-H bonds overlapped with the stretching of N-H bonds, and the peak at 2924 cm^−1^ and 2877 cm^−1^ is attributed to the stretching of C-H bonds. The peak at 1662 cm^−1^ corresponds to the amide I band, i.e., the stretching of the C=O group. The peak at 1571 cm^−1^ is attributed to the bending vibration of N-H bonds of NH_2_ and the CONHR group (the amide II band). The bands at 1417 cm^−1^, 1383 cm^−1^, and 1321 cm^−1^ are attributed to the bending of C-H bonds of the CH_3_, CH_2_, and CH groups, respectively. The absorption at 1259 cm^−1^ is associated with the stretching vibrations of the C-N bonds, whereas the peaks at 1156 cm^−1^ and 1080 cm^−1^ are attributable to the stretching of the C-O-C bonds (a glycosidic bond and a bond between anomeric C_1_ and O in the pyranose skeleton). The peak at 1025 cm^−1^ is characteristic of the stretching vibrations of the C-OH bonds in secondary and primary hydroxyl groups, while the band at 895 cm^−1^ is associated with the wagging of the saccharide skeleton of chitosan. The calculated DA% of the extracted chitosan was approximately 14%, resulting in a DDA% of around 86%.

### 3.3. Astaxanthin Identification and Quantification

Analysis through HPLC-HRMS confirmed the presence of astaxanthin in the ethanolic extract of *Procambarus clarkii*. The chromatogram displayed a peak with a retention time of 13.6 min, which can reasonably be attributed to astaxanthin-like structures, such as geometrical isomers, based on the observed *m*/*z* value in the high-resolution mass spectra (Δppm = −2.8) and the corresponding MS/MS spectrum.

**Figure 2:**
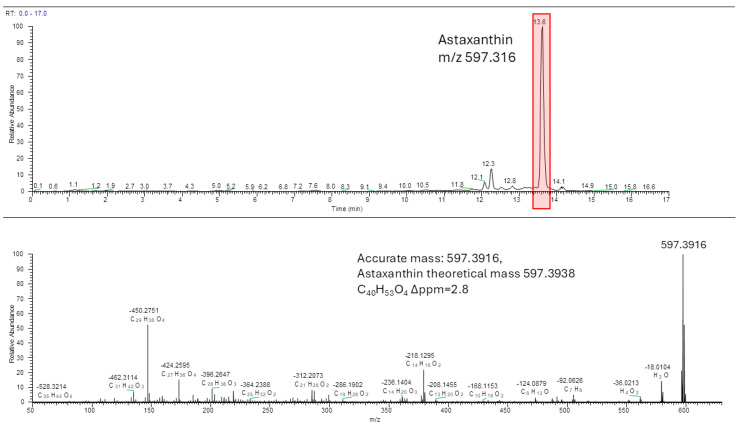
HPLC/MS chromatogram and MS/MS spectrum of the peak 13.6 min from the ethanolic *P. clarkii* extract.

The MS/MS spectrum, acquired under the same experimental conditions as those reported for astaxanthin in the publicly available MZCloud database, showed a fragmentation pattern that was superimposable onto the reference spectrum (Figure 2).

The concentration of astaxanthin present in the exoskeleton flour, measured in ethanolic extract using HPLC-HRMS analyses, was 581 ± 58 μg/g.

### 3.4. Polyphenol Compound Identification and Quantification

The TPC value calculated for the phenolic extract was 1.9 ± 0.04 mg GAE/g. The bio-phenol compounds identified through UHPLC-MS analysis are summarized in Table 2. Mandelic acid was detected in the highest concentration, while trans-hydroxycinnamic acid, ferulic acid, rutin, and p-coumaric acid were present in lower amounts. Gentisic acid and luteolin were also identified, although their concentrations were below the limit of quantification (LOQ).

**Table 2:** Bio-phenol compounds detected in *P. clarkii* exoskeleton.

Bio-Phenol	Calibration Equation	mg/100 mL
Mandelic Acid	Y = −25181.1 + 488.34X	1.5
Trans-OH-Cynnamic Acid	Y = −121.915 + 6685.59X	0.2
Ferulic Acid	Y = 1614 + 1181X	0.12
Rutin	Y = −61,324.9 + 2962.87X	0.1
Coumaric Acid	Y = −10,089 + 6127X	0.5
Gentisic	Y = −58,051.4 + 3441.27X	<LOQ
Luteolin	Y = −1577 + 10979X	<LOQ

### 3.5. Reducing and Radical Scavenging Capacity

The results of the reducing and radical scavenging assays are summarized in Table 3. Significant differences were observed in all reducing capacity and radical scavenger activity assays when comparing chitosan, astaxanthin, and phenolic compound samples. As expected, the greatest antioxidant activity was observed for all three assays in the astaxanthin samples (4.27 ± 0.24 mg AAE/g, 7.91 ± 0.19 µmol TE/g, and 9.76 ± 0.14 µmol TE/g for FRAP, DPPH, and ABTS assays, respectively). However, a difference was observed between the chitosan and phenolic extract; while chitosan showed greater reducing capacity in the FRAP assay (3.27 ± 0.14 mg AAE/g compared to the phenolic extract’s 1.21 ± 0.03 mg AAE/g), the phenolic extract exhibited significantly higher values in the radical scavenging assays DPPH and ABTS (4.93 ± 0.07 µmol TE/g and 6.89 ± 0.14 µmol TE/g, respectively, compared to the chitosan’s 2.22 ± 0.07 µmol TE/g and 3.82 ± 0.13 µmol TE/g).

**Table 3:** Reducing and radical scavenging of chitosan, astaxanthin and phenolic compounds extracted from *P. clarkii.* Values are the mean ± SD of three determinations carried in duplicate (ANOVA one-way associated with Tukey’s test). Values in the same column with different letters are significantly different at *p* < 0.05.

	FRAP mg AAE/g	DPPH^•^ µmol TE/g	ABTS^•+^ µmol TE/g
Chitosan	3.27 ± 0.14 ^c^	2.22 ± 0.07 ^c^	3.82 ± 0.13 ^c^
Astaxanthin extract	4.27 ± 0.24 ^b^	7.91 ± 0.19 ^b^	9.76 ± 0.14 ^b^
Phenolic extract	1.21 ± 0.03 ^a^	4.93 ± 0.07 ^a^	6.89 ± 0.14 ^a^

FRAP: Ferric Reducing Antioxidant Power; DPPH^•^: 2,2-diphenyl-1-picrylhydrazyl; ABTS^•^+: 2,2′-azino-bis (3-ethylbenzothiazoline-6-sulfonic acid); AAE: Acid Ascorbic Equivalent; TE; Trolox equivalent.

### 3.6. In Vitro Antitumoral Activity

**Figure 3:**
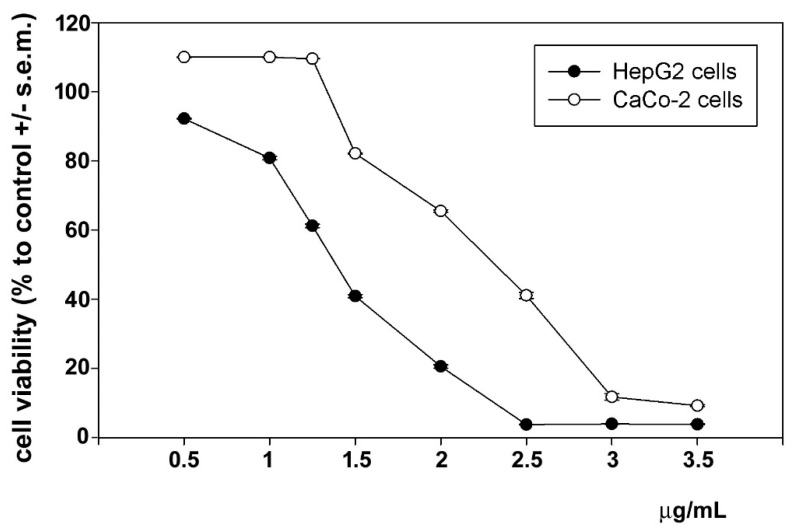
Dose–response effect of phenolic extract from *P. clarkii* exoskeleton on the viability of HepG2 and CaCo-2 cells after 24 h of exposure. The error bars correspond to the standard error of the mean (s.e.m.) of three independent measurements. *p* values comparing viability ratios to controls were <0.05 for every measurement.

**Figure 4:**
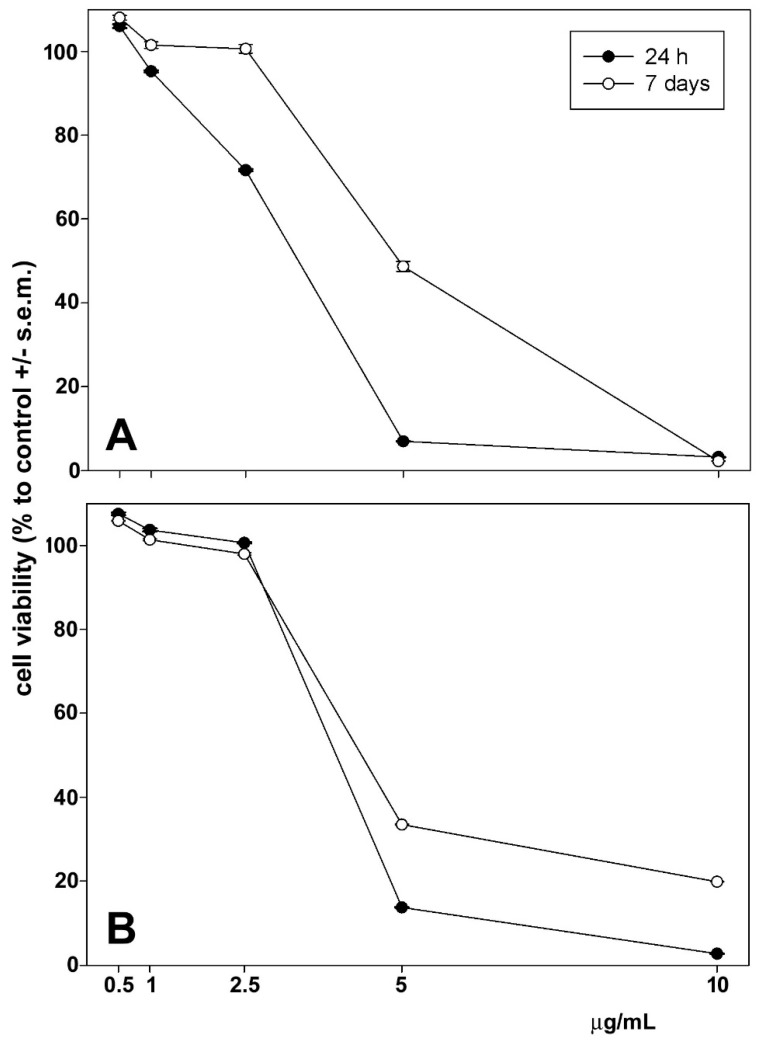
Dose–response effect of 24 h and 7 days of extraction of astaxanthin extract from *P. clarkii* exoskeleton on the viability of HepG2 (**A**) and CaCo-2 cells (**B**) after 24 h of exposure. The error bars correspond to the standard error of the mean (s.e.m.) of three independent measurements. *p* values comparing viability ratios to controls were <0.05 for every measurement.

The cytotoxic effects of astaxanthin and phenolic extract on HepG2 and CaCo-2 cells were examined using an MTT assay. Astaxanthin was administered to cells 24 h and 7 days following extraction. As shown in Figure 3 and Figure 4, cell exposure for 24 h to phenolic extract and 24 h to extracted astaxanthin resulted in a concentration-dependent decrease in cell viability, and the mean IC_50_ was estimated to be 1.45 µg (phenolic extract) and 4.28 µg (astaxanthin extract) of dry extract/mL for HepG2 cells and 2.45 µg (phenolic extract) and 4.57 µg (astaxanthin extract) of dry extract/mL for CaCo-2 cells. Interestingly, after 7 days from extraction astaxanthin did not show any significant modification of the mean IC_50_ values, estimated to be 4.89 µg (HepG2 cells) and 4.95 µg (CaCo-2 cells) of dry extract/mL, indicating the maintenance of the functional stability of the molecule within the considered time frame.

### 3.7. In Vitro Anti-Inflammatory Activity

**Figure 5:**
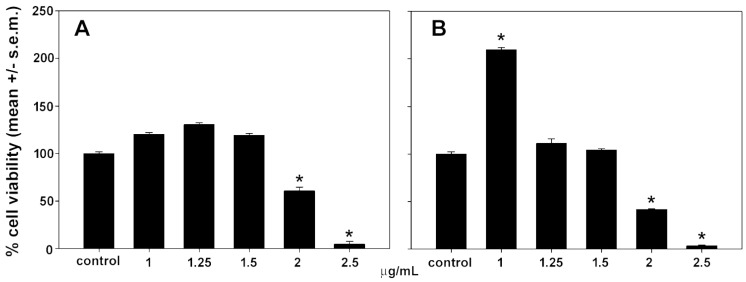
Effect of astaxanthin (**A**) and polyphenol extract (**B**) from *P. clarkii* exoskeleton on the viability of RAW 264.7 macrophages after 24 h of exposure. The error bars correspond to the standard error of the mean (s.e.m.) of three independent measurements. * *p* < 0.05.

**Figure 6:**
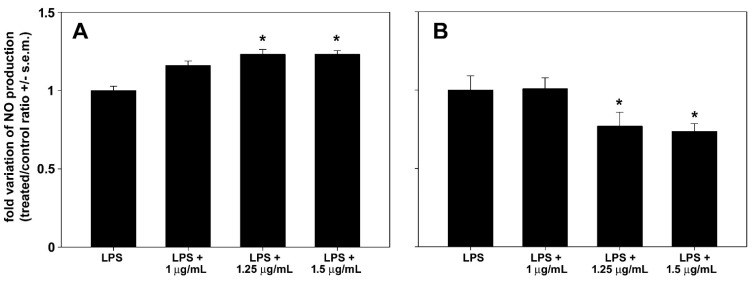
Effect of astaxanthin (**A**) and polyphenol extract (**B**) from *P. clarkii* exoskeleton on NO production by RAW 264.7 macrophages after 24 h of co-exposure with LPS compared with LPS alone. The error bars correspond to the standard error of the mean (s.e.m.) of three independent measurements. * *p* < 0.05.

LPS stimulation is known to trigger iNOS transcription and protein synthesis in RAW 264.7 macrophages cells, leading to NO production. Preparations that inhibit pathways leading to NO production are promising candidates for treating inflammatory diseases. In an initial set of assays, we selected the MNICs of astaxanthin and polyphenol extracts to be co-administered with LPS to macrophage cells. As shown in Figure 5, RAW 264.7 cell exposure to either astaxanthin or polyphenolic extract for 24 h resulted in a concentration-dependent decrease in cell viability starting at 2 μg/mL, with selected MNICs of 1, 1.25, and 1.5 μg of both dry extracts/mL. Notably, incubation with 1 μg of polyphenol extract/mL approximately doubled cell viability. As shown in Figure 6, no reduction in NO release was observed following co-treatment with LPS and either 1, 1.25, or 1.5 μg of astaxanthin/mL; instead, a slight increase of approx. 16%, 23%, and 23%, respectively, was observed. On the contrary, cell co-exposure to LPS and either 1.25 or 1.5 μg of polyphenol extract/mL determined a reduction in the treated/control ratio of NO to about 77% and 74%, respectively. The lowest concentration tested, i.e., 1 μg/mL, was ineffective in decreasing the LPS-induced NO production of cells.

## 4. Discussion

The presence of the invasive species *Procambarus clarkii* has been documented in Sicily since 2003 [52], while at the sampling site (Lake Rosamarina) it was first recorded in 2012 [53]; today, despite various efforts to eradicate the species, it remains widespread across numerous sites [52,53,54]. Among the possibilities for exploiting this species, the extraction of bioactive molecules represents a promising and widely studied frontier [2,3,4,15,16,38,39,40]. Given that the exoskeleton constitutes a significant portion of the total weight of Sicilian *P. clarkii* (87.84%), this study aimed to use this matrix to extract bioactive molecules (chitosan, astaxanthin, and bio-phenols) and evaluate their potential anticancer and anti-inflammatory activity, as well as their reducing capacity and radical scavenger activity.

### 4.1. Bioactive Molecules’ Characterization and Antitumoral and Anti-Inflammatory Activity

Chitin is a polysaccharide that performs a mechanical and structural function in the exoskeletons of crustaceans [55], and it was extracted from the ground exoskeleton of Sicilian *Procambarus clarkii*. Chitin was then deacetylated to obtain chitosan, with a yield of 12.29 ± 0.73% in its purified form. Chitosan can be extracted using chemical, biological, and biotechnological techniques [42]. The chitosan yield obtained in our study depends not only on the chitin content in the exoskeleton but is also highly dependent on the extraction technique and the reaction conditions [56]. The chitosan yield was similar to the yield extracted from Egyptian-farmed *P. clarkii* obtained by Taher et al. [2], in which the reaction conditions of the chitin deacetylation and the chitosan purification steps were the same. The chitosan extracted in our study showed a deacetylation degree of 86%, which is similar to results reported by Taher et al. [2], Omar et al. [57], and El-Naggar et al. [4] in chitosan extracted from Egyptian *P. clarkii*. The degree of deacetylation in chitosan depends on the chitin extraction method, and reaction conditions during deacetylation (alkali concentration, temperature, and time) [58] are key parameters influencing the chitosan’s physicochemical properties and biological activity [59].

From the exoskeleton of Sicilian *Procambarus clarkii*, using an ultrasound-assisted extraction technique and ethanol as the solvent, we obtained 581 ± 58 μg/g of astaxanthin. We obtained greater amounts than those reported by other authors who extracted astaxanthin from the exoskeleton of *P. clarkii* using both chemical [15,44] and biological techniques [14]. The variation in astaxanthin content extracted from crustacean exoskeletons is influenced by the extraction technique employed [60]. Recent studies have reported promising results with ultrasound-assisted extraction [61,62,63]. This technique utilizes ultrasonic waves to generate cavitation bubbles in the solvent, which have a mechanical impact on the exoskeleton powder particles. This process increases the contact area between the liquid and solid phases, allowing for greater solvent penetration into the matrix and improving extraction efficiency [62]. Moreover, the amount of astaxanthin extracted also depends on the carotenoid levels in the raw material. In crustaceans, this parameter is highly variable, depending on factors like the season of capture, the habitat, feeding habits, the reproductive cycle, and the molting stage [64,65].

We tested the cytotoxic ability of astaxanthin obtained from *Procambarus clarkii* against two human cancer cell lines, HepG2 from hepatocarcinoma and CaCo-2 from colorectal cancer, and found a dose-dependent effect in both cell models. These results are consistent with literature reports on other crustaceans species, demonstrating the antitumoral activity of astaxanthin present in the by-products of *Parapenaeus longirostris* shrimp and in krill oil on the same cells [66,67].

Phenolic compounds represent another class of molecules that could add value to the exoskeletons of Sicilian *Procambarus clarkii*. Our results showed that exoskeleton extracts contain a range of bioactive compounds, including phenolic compounds identified in methanolic extracts. Polyphenols, one of the most widespread classes of secondary metabolites in nature, primarily originate from the amino acids phenylalanine and tyrosine. These amino acids undergo deamination to form cinnamic acids, which subsequently enter the phenylpropanoid pathway [68]. The presence of phenolic compounds in *P. clarkii* is suggested by the presence of the enzyme phenoloxidase [69,70], for which phenols act as substrates [71]. This study investigated TPC in the methanolic extracts of exoskeleton of this species. The TPC was 1.9 ± 0.04 mg GAE/g. Comparable results were obtained by Elkhodary et al. [38], who previously evaluated TPC on aqueous and methanolic extracts of *P. clarkii* exoskeletons captured in the River Nile. Furthermore, our TPC results were higher than those of Onodenalore et al. [21] on ethanolic extracts of *Pandalus borealis* (flesh: 0.19 ± 0.04 mg GAE/g; shells: 0.28 ± 0.04 mg GAE/g; heads: 0.15 ± 0.04 mg GAE/g; whole shrimp: 0.21 ± 0.02 mg GAE/g). In contrast, our results were lower than those obtained by Pereira et al. [25] on aqueous and ethanolic extracts of the exoskeleton of *Litopenaeus vannamei* (3060.06 ± 1.84 mg GAE/100 g; 1993.26 ± 94.42 mg GAE/100 g) and *Ucides cordatus* (1623.24 ± 48.07 mg GAE/100 g; 1131.11 ± 49.95 mg GAE/100 g), and those obtained by Maia et al. [72] on ethanolic extracts of exoskeletons of *Palaemon serratus* (10.4 ± 0.7 mg GAE/g at 6.4 ± 0.4 mg GAE/g) and *Palaemon varians* (9.9 ± 0.6 mg GAE/g at 4.7 ± 0.3 mg GAE/g). It was observed that the species, location, capture season, and extraction technique can significantly influence the TPC of phenolic extracts from crustaceans [25,70]. Given that the phenolic compound content in crustaceans could be correlated with their diet [21], differences in TPC between our study and results from previous studies could be due to variations in habitat. Among the identified phenolic compounds, five were above the limit of quantification (LOQ): p-coumaric acid, mandelic acid, trans-hydroxycinnamic acid, rutin, ferulic acid, and luteolin.

These compounds exhibit a broad spectrum of biological activities commonly associated with their antioxidant, anti-inflammatory, antimutagenic, antimicrobial, and antibacterial growth-inhibiting properties. The literature also reports that phenolic compounds have algicidal, antifungal, insecticidal, and estrogenic activities, potentially serving as protective mechanisms for the organism within its biological environment [22,23,38]. Mandelic acid, in particular, is an aromatic alpha hydroxy acid [73], and it has antimicrobial effects. It is the most abundant of the identified bio-phenols in the ground exoskeletons, all of which are characterized by antioxidant, anti-inflammatory, and protective effects. Our findings demonstrate that polyphenols from *P. clarkii* reduce tumor cell viability and suppress the inflammatory response in stimulated macrophages. According to data in the literature, most constituents of the polyphenol preparation are likely responsible for the observed cytotoxic and anti-inflammatory effects. Regarding anti-tumoral activity, p-coumaric acid-rich extracts from various edible mushrooms have been shown to affect HepG2 cell viability [74]. In addition, p-coumaric acid treatment was found to inhibit the proliferation of CaCo-2 cells by influencing the expression of cell-cycle-regulating genes and increasing the cell fraction in the subG_1_ and G_2_ phases [75,76]. Ferulic acid was shown to disrupt the cell cycle, induce mitochondrial depolarization, and promote apoptosis in HepG2 cells while also up-regulating biochemical markers of apoptosis and autophagy [77]. The p-coumaric-acid- and ferulic-acid-rich immature wheat bran extracts were powerful inhibitors of CaCo-2 cell proliferation [78]. Rutin and luteolin were found to stimulate autophagy in HepG2 cells, with the former also exerting a cytotoxic effect on CaCo-2 cells [79,80,81]. Gentisic-acid-rich pistachio extracts exhibited strong anti-proliferative activity on HepG2 and CaCo-2 cells [82]. Regarding the observed NO production inhibition in inflamed RAW 264.7 macrophages, our findings are consistent with those of Shen et al. [83], which demonstrated that coumaric-acid- and rutin-rich extracts from jujube peel down-regulated ROS, NO, prostaglandin E_2_, and inflammatory cytokines by inhibiting the MAPK and NF-κB pathways. Concurrently, they promoted the expression of Nrf2 protein and the secretion of glutathione peroxidase, superoxide dismutase, and catalase. Similarly, luteolin inhibited the production of NO and prostaglandin E_2_ as well as the expression of their corresponding enzymes, iNOS and cyclooxygenase-2 [84]. Furthermore, ferulic acid was found to reduce NO production in inflamed RAW 264.7 cells through the phosphorylated IKK decrease-mediated down-regulation of NRF-2 and NF-κB translocation into the nuclei, thereby inhibiting the transcription of genes coding for inflammatory mediators [85]. Rutin and gentisic acid were also effective in decreasing the expression of iNOS and exerting an anti-inflammatory effect on LPS-stimulated cells [86,87]. Some bio-phenols (rutin) have been associated with anti-cancer effects [88,89]. Both ferulic acid and p-coumaric acid possess strong antioxidant and anti-inflammatory properties capable of neutralizing free radicals and safeguarding cells from oxidative stress. These compounds have been studied for their potential antitumor effects and as chelating agents [90].

### 4.2. Reducing and Radical Scavenging Capacities

Chitosan, astaxanthin, and phenolic compounds are known to possess important antioxidant activities. The antioxidant activity of chitosan is mainly due to the hydroxyl groups (-OH) in position C3 and C6 and the amino group (-NH_2_) in C2 of the glucosamine unit [91]. The antioxidant activity of chitosan has also been shown to increase with a higher degree of deacetylation [92]. Astaxanthin’s exceptional antioxidant capacity is attributed to its unique structure, featuring a central polyene chain, which can donate electrons [93], and keto (C=O) and hydroxyl (-OH) groups at each end capable of donating hydrogen atoms [14]. Phenolic compounds owe their antioxidant activity primarily to hydroxyl groups, which, by donating hydrogen atoms to the free radicals, prevent their propagation phase. In fact, it has been observed that their antioxidant activity is proportional to the number of hydroxyl groups on the aromatic ring(s). The position of the hydroxyl groups is also crucial for antioxidant activity, as these can chelate pro-oxidative metals, activate antioxidant enzymes, and form antioxidant-active adducts [94]. Based on this, the reducing and radical scavenging activities of the extracted and characterized bioactive molecules were evaluated using FRAP, ABTS, and DPPH assays. The FRAP assay evaluates the ability of the molecules to donate electrons to reduce the ion Fe^3+^ (iron III) to Fe^2+^ (iron II) [50]. The DPPH [50] and ABTS [49] assays measure the scavenging activity of the molecules against DPPH^•^ and ABTS^•+^ radicals, respectively, through electron or hydrogen atom transfer. Although previous studies have examined the reducing and radical scavenging activities of chitosan and astaxanthin extracted from the exoskeleton of *Procambarus clarkii* [14,37,57,95], to our knowledge, no studies have investigated the antioxidant activity of phenolic extracts from this species.

This study is the first to conduct three different tests to evaluate the reducing and radical scavenging potential of chitosan, astaxanthin, and phenolic compounds extracted from the exoskeleton of *P. clarkii* while comparing the results to identify any significantly superior activities. Our findings showed that all of the molecules tested exhibit antioxidant activity both in terms of reducing capacity and free radical scavenging activity. All of the assays revealed significantly different activity between the bioactive molecules tested. Astaxanthin showed the greatest activity in all assays, chitosan showed a higher reducing capacity than the phenolic extract in the FRAP assay, and the phenolic extract exhibited greater radical scavenging activity than chitosan in the DPPH and ABTS assays. Our results demonstrate strong radical scavenger activity of astaxanthin in the DPPH assay consistent with findings obtained by Hamdi et al. [14] and Elkhodary et al. [38] on astaxanthin extracted from the exoskeleton of *P. clarkii* captured in the River Nile. Moreover, the results obtained for the DPPH assay performed on chitosan are similar to those of Omar et al. [57] and Makkey et al. [95] on chitosan extracted from the exoskeleton of Egyptian *P. clarkii*.

## 5. Conclusions

This study highlights the potential of *Procambarus clarkii* exoskeletons as a source of bioactive molecules, such as chitosan, astaxanthin, and bio-phenols. All extracted compounds demonstrated reducing and radical scavenger activities. In particular, chitosan, due to its high purity and elevated DDA%, could find applications in the biomedical field. Additionally, astaxanthin and polyphenol extracts showed antitumor effects on hepatocellular carcinoma (HepG2) and colorectal cancer (CaCo-2) cell lines, suggesting the need for further studies to explore their potential in cancer treatment. In LPS-stimulated RAW 264.7 macrophages, polyphenols reduced nitric oxide release, indicating promising anti-inflammatory activity that warrants further investigation. These results are particularly significant considering that chitosan is currently mainly extracted from crustacean shells and astaxanthin from microalgae, with both methods involving high costs and significant environmental impacts. The use of *P. clarkii* exoskeletons, an invasive species abundant in the Mediterranean, could offer numerous benefits, including contributing to local population control of this species. After optimizing the extraction methods, this new resource could reduce production costs without harming natural ecosystems. Furthermore, using *P. clarkii* for the extraction of chitosan and astaxanthin could represent an eco-friendly and sustainable solution compared to traditional sources, transforming an ecological challenge into a biotechnological opportunity for the pharmaceutical, cosmetic, and nutraceutical sectors. Future studies could focus on optimizing extraction methods to increase yields and bioactivity, as well as exploring the synergistic effects of these bioactive molecules in pharmaceutical applications.

## Data Availability

Data will be provided upon request.
